# Effect of Climate-Related Change in Vegetation on Leaf Litter Consumption and Energy Storage by *Gammarus pulex* from Continental or Mediterranean Populations

**DOI:** 10.1371/journal.pone.0077242

**Published:** 2013-10-18

**Authors:** Natacha Foucreau, Christophe Piscart, Sara Puijalon, Frédéric Hervant

**Affiliations:** 1 UMR 5023 Ecologie des Hydrosystèmes Naturels et Anthropisés; Université de Lyon; Université Lyon 1; ENTPE; CNRS; Villeurbanne, France; Institute of Marine Research, Norway

## Abstract

As a consequence of global warming, it is important to characterise the potential changes occurring for some functional processes through the intra-specific study of key species. Changes in species distribution, particularly when key or engineer species are affected, should contribute to global changes in ecosystem functioning. In this study, we examined the potential consequences induced by global warming on ecosystem functioning in term of organic matter recycling. We compared consumption of leaf litter by some shredder populations (*Gammarus pulex*) between five tree species inhabiting continental (i.e., the northern region of the Rhône River Valley) and/or Mediterranean (i.e., the southern region of the Rhône River Valley) conditions. To consider any potential adaptation of the gammarid population to vegetation in the same climate conditions, three populations of the key shredder *Gammarus pulex* from the northern region and three from the southern region of the Rhône River Valley were used. We experimentally compared the effects of the geographical origin of both the gammarid populations and the leaf litter species on the shredding activity and the physiological state of animals (through body triglyceride content). This study demonstrated that leaf toughness is more important than geographical origin for determining shredder leaf litter consumption. The overall consumption rate of the gammarid populations from the southern region of Rhône Valley was much higher than that of the populations from the northern region, but no clear differences between the origins of the leaf litter (i.e., continental *vs*. Mediterranean) were observed. The northwards shift of *G. pulex* populations adapted to warmer conditions might significantly modify organic matter recycling in continental streams. As gammarid populations can demonstrate local adaptations to certain leaf species as a trophic resource, changes in riparian vegetation associated with climate change might locally affect the leaf litter degradation process by this shredder.

## Introduction

Aquatic fauna will have to deal with increasing temperatures [1], [2], [3], ranging from +2°C to +6°C for atmospheric temperatures [4] which are strongly correlated with water temperatures. In response to climate change, the geographical distribution of some species is currently shifting [5]. Indeed, the environmental temperature influences latitudinal species distribution patterns and species replacements [6], [7], [8]. This shift, particularly when key or engineer species are affected, contributes to global changes in ecosystem functioning [9]. Consequently, functional processes within habitats might be altered due to the local disappearance of species populations [8].

Most studies concerning the thermal gradient in the context of climate change involve several species [10], [11], [12], [13], [14], [15]. However, few studies have focused on the consequences of climate change on different populations of the same species [16], [17], whereas the potential adaptation of populations inhabiting the margin of the distribution area, particularly of species with a wide distributional area, might greatly modify the response at the species level [7], [18]. For widely distributed species, characterising changes at the intra-specific level could be crucial to highlight the potential effects of climate changes.

Climate changes are also associated with changes in the distribution of vegetation species [19], [20], [21], which might significantly modify the availability of feeding resources in terrestrial and adjacent aquatic ecosystems, because the consumption of the leaves of riparian trees by the shredder species constitutes a major allochtonous trophic resource for temperate stream ecosystems [22], [23]. In addition, the shredder leaf consumption rate is influenced by leaf characteristics [24], [25]. Therefore, the potential replacement of temperate vegetation with Mediterranean species [20] showing different biomechanical and/or chemical characteristics (tannin composition, wax components) might significantly alter ecosystem function [26], [27].

The aim of this study was to experimentally measure the potential consequence of the climate-related vegetation change on leaf litter consumption by the key freshwater shredder species, *Gammarus pulex* [28], [29]. To consider any local adaptation of the shredder populations, six populations of *G. pulex* were used (i.e., three populations from the northern region of the Rhône Valley and three from the southern region), feeding on either northern continental vegetation or southern Mediterranean vegetation. Four leaf litter species were selected as two pairs of phylogenetically closely related species. Each pair comprised one species adapted to the northern continental climate from the North of the Rhône River Valley and one species adapted to the southern Mediterranean climate from the South of the Rhône River Valley. As leaf toughness, which is related to the leaf mechanical structure, significantly modifies shredder feeding activity, we studied two pairs of leaf species: two species of hornbeam with soft leaves and two species of oak leaves with tough leaves [27]. A fifth species, present in both the southern and northern regions of Rhône Valley, with soft leaves was also studied. Thus, we compared the effects of the geographical origins of both animals and plants on the consumption rate of gammarid populations and on their physiological state (body triglyceride content) over a 10-day period. We assumed a 50∶50 ratio between males and females for our replicates.

We proposed the following hypotheses: (i) the Mediterranean leaf litter species adapted to dry environmental conditions [30], is harder to consume (due to higher mechanical resistance of leaf tissues) than northern leaf litter species [31], [32], [33]; (ii) as southern populations of *G. pulex* are exposed to tougher leaves in their natural environment, these species should adapt to consume harder leaves and therefore consume more southern leaves (and northern leaves, which are easier to consume) than the northern populations of G. *pulex*; and (iii) the amount of food consumed is not necessarily associated with the efficiency of energy storage in animals [34], [35], because leaves of different species might vary in nutritional quality [32], [36], [37].

## Materials and Methods

Sampling sites were located in public areas and did not require specific permission to sample; no endangered or protected species were involved in these areas and no vertebrates were used for this study.

### 2.1. Selection and conditioning of tree leaves

This study was performed using five leaf litter species according to their adaptation either to continental or Mediterranean climates. Three *Betulaceae* species with relatively soft leaves were used: alder leaves (*Alnus glutinosa* L. Gaertn.), commonly found along the streams and rivers in continental climates and locally in Mediterranean climates [38], [39]; European hornbeam leaves (*Carpinus betulus L.*) occurring in continental climates [40], [41] and widely distributed throughout the northern regions of the Rhône River Valley; and European Hop-hornbeam (*Ostrya carpinifolia L.*) inhabiting Mediterranean conditions in the southern regions of the Rhône River Valley [42]. Two oak species with tough leaves [43] were also used: Pedunculate Oak (*Quercus robur L.*) from the northern regions of the Rhône Valley [20] and Downy Oak (*Quercus pubescens Wild*) [22], inhabiting Mediterranean conditions. Leaves of the continental species *A. glutinosa* and *Q. robur* were collected from the same stand of trees near Lyon, France (45°49′36′′N, 05°04′53′′E) and *C. betulus* hornbeam leaves were collected from a separate but nearby location (45°49′36′′N, 05°04′53′′E). The leaves of the Mediterranean species *O. carpinifolia* were collected near Nice, France (43°47′59.05′′N, 7°24′33.25′′E), and *Q. pubescens* leaves were collected near Toulon (43°31′11.04′′N, 7°32′36.4′′E). In autumn, three hundred fallen leaves were collected for each species and air-dried.

In the winter, disks from each leaf species were stream-conditioned in the same water body by the same pool of hyphomycete species under natural conditions, according to the description of Foucreau *et al.* [27]. As this study monitored the consumption rate of leaf litter by shredders, leaves had to be conditioned for long enough so that gammarids consumed enough leaves to obtain a consumption rate. Due to initial differences in leaf toughness and in the conditioning rate, alder leaves were conditioned for 10 d, whereas hornbeam leaves were conditioned for 25 d, and oak leaves for 35 d [27]. Before and at the end of the conditioning, the biomechanical characteristics and leaf thickness were measured according to Dehedin *et al.* [44] and Foucreau *et al.* [27]. The leaf thickness (mm) was measured using a digital thickness gauge (±0.01 mm), avoiding major veins. To determine the leaf toughness (J m^−2^), we used punching tests, which measure the force required to punch a hole through the leaf lamina using a universal testing machine (Instron 5942; Instron SAS, Canton, MA, USA). Toughness was calculated as the force required to punch a hole through the leaf lamina, corrected by the area of the punch. After conditioning, the ergosterol content was measured, to assess the fungal biomass [45]. Three replicates of leaf material for each species were freeze-dried (Christ® ALPHA 1–4LD) and weighed to the nearest 0.1 mg. For each replicate, ergosterol was extracted twice successively with methanol (25∶1 v/w) for 15 min, followed by 15 min of sonication and ergosterol was quantified by HPLC DAD (Agilent 1200 series), according to the method of Dehedin *et al.* [44].

### 2.2. Selection, sampling and rearing of *Gammarus pulex* populations

**Figure 1:**
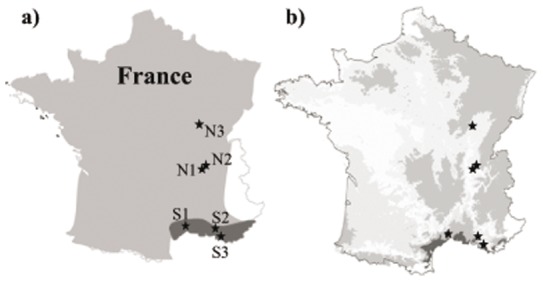
Geographical distributions of G. pulex and G. pulex gallicus, and mean annual temperatures in France. Map of France with (a) the geographical distribution of Gammarus pulex shown in light gray, and the geographical distribution of Gammarus pulex gallicus (subspecies) shown in dark gray (adapted from [44]); and (b) mean annual temperatures (from WorldClim, Global Climate Data, http://www.worldclim.org/), from low (light gray) to high temperatures (dark gray) (categorization). The gammarid sampling sites are indicated with black stars: S1 (03°47′22′′E, 43°39′3.4′′N), S2 (05°23′57′′E, 43°34′42′′N) and S3(05°36′13′′E, 43°18′15′′N); N1 (05°06′23′′E, 45°49′35′′N), N2 (04°53′0.9′′E, 47°24′13′′N) and N3 (05°13′51′′ E, 45°50′06′′ N).

The amphipod *Gammarus pulex* (Linnaeus 1758) is the most widely distributed amphipod species in Europe [46] and plays an essential role in leaf litter recycling in streams [29], [47]. In the Rhône River Valley (Fig. 1a), *G. pulex* is distributed from the northern (temperate continental climate) to the southern (Mediterranean climate) regions (Fig. 1b). Moreover, the sub-species *G. pulex gallicus* populations (Karaman 1931) in the Southern Rhône River Valley are morphologically [46] and physiologically [18] different from the northern populations. These studies suggested a higher metabolism and locomotory activity for the southern populations compared to the northern ones, which might influence the feeding activity. Three populations of *G. pulex* were sampled from the northern region of the Rhône River Valley (N1, N2, and N3), and three *G. pulex gallicus* populations were sampled from the southern region (S1, S2, and S3) (Fig. 1). For each region, the collecting sites were similar in terms of riparian vegetation cover (mainly alder and oak leaves in the north and oak leaves in the south), substratum type, width, and current velocity. At least 125 pre-copula pairs from each population were sampled in January 2012 (a total of 1,500 individuals) using a hand-net sampler. The female and male individuals of each pair were carefully separated directly in the field. To verify that the populations of the different sites belonged to the same species, DNA of 10 animals from each population was amplified with primers targeting the mitochondrial cytochrome oxidase subunit I (COI) gene (data not shown) and the threshold method defined by Lefébure *et al.* [48] was applied to verify that patristic distances computed from the phylogeny were much lower than the 16% divergence threshold between two different species [48]. The animals were acclimated to laboratory conditions for 7 d in 5-L tanks filled with aerated water obtained from their own site at 12°C, under a 12:12 h light:dark cycle and provided with crustacean food (JBL NovoCrabs®) *ad libitum* to standardise the physiological conditions of these animals. We assumed that the variation in the water physico-chemistry among sites had less influence on the consumption rate by gammarids than the population or vegetation effects.

### 2.3 Shredder leaf consumption

After acclimation, the animals of each population were placed individually in plastic cups (Ø7 cm) containing 60 mL filtered water (Whatman™, GE Healthcare Companies, UK, Ø20–25µm) from their own sampling site. Shredder leaf consumption was examined using a full factorial design with three factors (animal sex, animal population of origin and leaf species) and n = 20 animals per condition. To characterise the differences in energy storage between males and females, the leaf consumption rate and body triglyceride content were measured separately for each sex. For the five tree species, three disks that were cut and then weighed (Ø16 mm) from the conditioned leaves of the same tree species, were added to each cup to reduce the intra-specific variability in the leaf thickness or toughness. After 10 d, the remaining leaf material was removed from the cups and the animals were removed after two d of starvation (i.e., after 12 d) to ensure that the presence of metabolites (from food) in the digestive bolus would not affect the triglyceride content [44]. The leaf material and animal samples were freeze-dried (Christ® ALPHA 1–4 LD), and the difference between the initial and the final dry leaf mass was used to compute the leaf consumption rate. Mass losses due to factors other than consumption (e.g., microbial growth and activity, leaching) were estimated using disks (N = 12 per tree species) incubated under the same conditions without animals. The control leaf disks, for which the final fresh and dry masses were determined, were also used to calculate a relationship between the fresh and dry masses. Hence, initial fresh masses of the disks were converted into initial dry masses and these initial dry masses were compared to the final dry mass of the disks to compute the leaf consumption rate. The leaf consumption rates were calculated as:
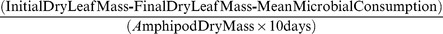



The leaf consumption rate was expressed in mg leaf dry mass day^−1^ mg^−1^ of gammarid dry mass [26].

In addition, this study characterised the energetic state of the individuals (through the triglyceride stores) after nutritional experimentation. In crustaceans, triglycerides constitute an essential compound for long-term energy storage [49], [50], [51]. Thus, individuals with a null leaf consumption rate (i.e., starved animals) were removed from the dataset concerning changes in the triglyceride content (TG). Indeed, the starved animals consumed their triglyceride stores to generate the energy necessary for metabolism. Eight animals per tree species per gender and per animal population were freeze-dried (Christ® ALPHA 1-4LD) and subsequently weighed. To measure the total triglyceride content, animals were homogenised in 3 mL of a chloroform:methanol mixture (2∶1, v/v) using an ultraturrax (Janke & Kunkel, IKA-WERK). The total TGs were measured using a standard enzymatic method with specific test combinations, according to Hervant *et al.* [48] and Hervant & Renault [52]. Specific triglyceride kits (GPO Trinder) were purchased from Sigma-Aldrich (France). All assays were performed using a spectrophotometer (Thermo Electron Corporation, AQUAMATE) at 25°C. For each condition, we determined the percentage variation in the mean body triglyceride content (TGs) (mg glycerol g^−1^ of dry gammarids) in *G. pulex* compared with the control animals freeze-dried before experimentation. For the calculation of these percentages, animals which presented a null leaf consumption rate were not taken into account.

### 2.6 Statistical analyses

Differences between leaf species with regard to leaf thickness and toughness were assessed before (at T0) and after conditioning, using two different one-way ANOVAs, with the leaf species as a factor. The ergosterol contents measured after conditioning were tested using a one-way ANOVA, with leaf species as a factor. As the gender factor showed a significant effect, we used a mixed model ANOVA for each sex to test the effect of the geographical origin of both vegetation and animals, with the leaf species and geographical origin (North or South) as factors, and the gammarid population factor nested within the geographical origin factor. Differences in the mean triglyceride content between females and males were tested using two-way ANOVAs with gender and gammarid populations as factors. For all ANOVAs, Tukey's HSD tests were performed for pairwise comparisons.

The linear correlations between the mean leaf consumption rate of northern or southern gammarid populations and the mean leaf species toughness were also tested. For each tree species, Spearman correlations were used to test the relationship between the mean percentage variation in the triglyceride content in gammarids and the leaf consumption rates. For this correlation, animals with null consumption rates were removed from the data set to the calculation of the leaf consumptions rates. All tests were performed using STATISTICA 7.1 software (Statsoft™, Tulsa, USA).

## Results

### 3.1. Characteristics of the initial and conditioned leaves

Before conditioning, thickness and toughness significantly differed between leaf litter species (Table 1; F_4, 143_  = 97.2, *p*<0.001 and F_4, 143_  = 90.4, *p*<0.001). Hornbeam leaves were the thinnest leaves, whereas *Q. pubescens* leaves were the thickest leaves (Table 1). The toughness of *A. glutinosa, C. betulus,* and *O. carpinifolia* leaves did not differ (*p*>0.05) but was consistently lower than that of *Q. robur* and *Q. pubescens* (*p*<0.001). In addition, the leaf toughness was higher for *Q. pubescens* than for *Q. robur* (*p*<0.001).

**Table 1:** Characteristics of the five leaf litter species before and after conditioning.

Geographical origin	North/South	North	South	North	South
Leaf litter species	*Alnus* *glutinosa*	*Carpinus betulus*	*Ostrya carpinifolia*	*Quercus robur*	*Quercus pubescens*
**Mean ergosterol** **contents** **(mg.g** ^−**1**^ **) ± SD**	1.98±0.80^a^	1.24±0.32^a, b^	1.60±0.26^a^	0.16±0.023^b^	0.37±0.12^b^
**Toughness** **(J.m** ^−^ **^2^)** ± **SD** Before conditioning After conditioning	189.0±45.1^a^ 182.8±51.5^a^	168.7±40.1^a^ 145.9±29.5^a, b^	160.3±27^a^ 110.9±27^b^	356.5±101.4^b^ 335.8±103^c^	577.3±189.7^c^ 457.4±192.8^d^
**Thickness (mm)** ± **SD** Before conditioning After conditioning	0.19±0.03^a^ 0.18±0.03^a^	0.11±0.01^b^ 0.12±0.01^b^	0.15±0.02^c^ 0.16±0.02^c^	0.20±0.03^a,d^ 0.19±0.03^d^	0.24±0.04^e^ 0.23±0.03^a, e^

Significant differences between leaf litter species are indicated by different letters.

After conditioning, the thickness, toughness, and ergosterol content were significantly different between the leaf litter species (Table 1; F_4, 146_  = 69.1, *p*<0.001; F_ 4, 146_  = 61.4, *p*<0.001, and F_4, 9_  = 8.7, *p* = 0.0037). The toughness and ergosterol content did not differ between the two hornbeam species (*p* = 0.67 and *p* = 0.99), but leaves of *O. carpinifolia* were significantly thicker than *C. betulus* leaves (*p*<0.001). The thickness of *A. glutinosa* leaves significantly differed from that of other species (*p*<0.001), except for *Q. robur* leaves (*p* = 0.99). The ergosterol content of *A. glutinosa* leaves did not differ from that of *O. carpinifolia* (*p* = 0.63) and *C. betulus* (*p* = 0.43) leaves, but was significantly higher than that of *Q. robur* and *Q. pubescens* leaves (*p*<0.01).

### 3.2 Leaf litter consumption rates

At the end of the experiment, the survival rates of gammarids were high (with only 9.0% mortality for northern gammarids and 7.3% for southern gammarids). The percentage of gammarids showing a null leaf consumption rate (i.e., starved animals) was low for southern populations (barely 9%) and higher for northern populations (almost 66%). Among the 66% of starved animal in northern populations, the percentages of animals that did not consume leaf material were similar among the five leaf species, ranging from 20.8 to 28.1%, except for *A. glutinosa* leaves, for which only 7% of animals did not consume leaves.

#### Leaf consumption rates of northern vs. southern leaf species

**Figure 2:**
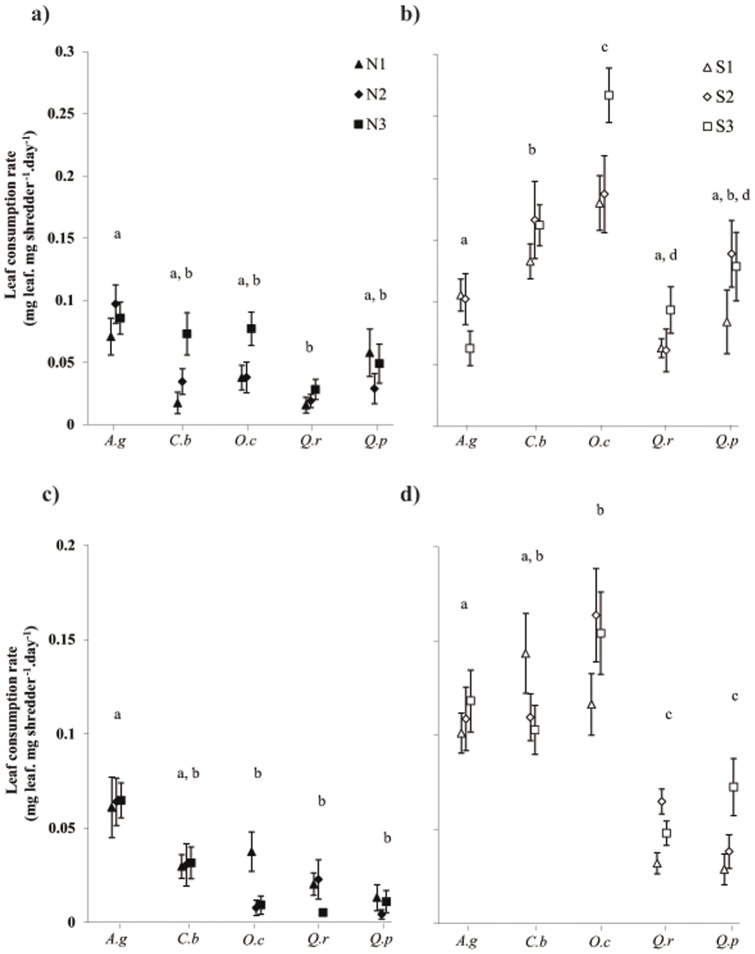
Leaf litter consumption rates. Mean leaf consumption rates (mg.d-1.g^−1^) ± SE of: (a) northern females,(b) southern females, (c) northern males, and (d) southern males for the five litter species, A.g (Alnus glutinosa), C.b (Carpinus betulus), O.c (Ostrya carpinifolia), Q.r (Quercus robur), Q.p (Quercus pubescens). Letters indicates significant differences (p<0.05).

The leaf consumption rates of both hornbeam leaves (*C. betulus vs. O. carpinifolia*) did not differ between all gammarid populations (*p*>0.05), regardless of the gender (0.087±0.004 *vs.* 0.10±0.007 mg leaf dry mass d^−1^ mg^−1^ of gammarid dry mass), except for females in the S3 population (Fig. 2, *p* = 0.004). Similarly, the leaf consumption rates of *Q. robur* (0.040±0.003) and *Q. pubescens* (0.053±0.005) leaves did not differ (*p*>0.05).

#### Leaf consumption rates of gammarid populations of the same geographical origin

We did not observe inter-population differences for the same geographical origin of gammarids of either sex (*p*>0.25), except for females of the S3 population, which significantly differed from the females of the S1 and S2 populations (F_4, 520_  = 3.12, *p* = .014). Globally, the populations from the same geographical origin and the same gender (North or South and females or males) showed similar patterns of leaf consumption rates (Fig. 2). For northern gammarid populations (Fig. 2a, c), we observed the same decreasing gradient of leaf consumption rate for males and females: *A. glutinosa  = C. betulus  = O. carpinifolia* >*Q. robur* and *Q. pubescens*. For southern females (Fig. 2b), the leaf consumption rate decreased in the order: *O. carpinifolia >C. betulus >A. glutinosa, Q. pubescens* and *Q. robur.* For southern males, the leaf consumption rate decreased from: *O. carpinifolia*  = *C. betulus >A. glutinosa >Q. robur* and *Q. pubescens* leaves (Fig. 2d).

The leaf consumption rates of females were significantly higher than those of males in the northern (+71%) and southern (+40%) populations (Fig. 2, F_3, 530_  = 11.78, *p*<0.001 and F_3, 539_  = 9.82, *p*<0.001, respectively).

In addition, a significant negative correlation between the mean leaf toughness and species consumption rates was observed for both northern and southern gammarid populations (*p* = 0.037 and *p*<0.001, respectively).

#### Leaf consumption rate of southern vs. northern gammarid populations

The overall leaf consumption rates of southern gammarids were higher than those for northern gammarids for the Mediterranean leaves of both females (i.e., +338% for *O. carpinifolia,* +144% for *Q. pubescens*; *p*<0.001) and males (+678% for *O. carpinifolia* and +422% for *Q. pubescens*, *p*<0.008). Concerning the consumption rate of continental leaves, *C. betulus* leaves were consumed more by southern gammarid populations (females and males) than by northern gammarid populations (females and males) (+275%, *p*<0.001; +287%, *p*<0.001, respectively), whereas the consumption rate of *Q. robur* leaves was significantly higher for females in the southern populations (+250%, *p* = 0.018) and not for males (*p* = 0.12). The consumption rate of *A. glutinosa* leaves did not differ for females (*p* = 0.99), but was slightly higher for southern than for northern males (+7.5%, *p*<0.001).

### 3.3 Leaf litter consumption and the triglyceride content of gammarids

The percentage variation in mean body triglyceride content changed after feeding, compared with the values of the control animals at T0, regardless of the leaf species and population. However, no clear pattern was observed in the populations or leaf species (Table 2). For each leaf species, we did not observe any correlations between the leaf consumption rates and the percentage variation in the triglyceride content of the gammarids for any leaf species, except for *A. glutinosa* of the northern gammarid population, for which the triglyceride content was negatively correlated (Table 3).

**Table 2:** Mean variation in the triglyceride content (% of the triglyceride content in mg.g−1 dry weight in control animals at T0) per sex and per gammarid population (S1, S2, S3, N1, N2, N3) for each leaf species (*A. glutinosa, C. betulus, O. carpinifolia, Q. robur, Q. pubescens*).

		S1	S2	S3	N1	N2	N3
Control at T0	Females	32	52	58	61	90	52
	Males	32	37	34	43	66	34
*Alnus* *glutinosa*	Females	56%	−22%	−20%	−41%	−48%	−15%
	Males	−7%	27%	−7%	−59.%	−29%	−30%
*Carpinus* *betulus*	Females	29%	−32%	−36%	−28%	4%	−43%
	Males	−19%	37%	−53%	−59%	−60%	−63%
*Ostrya* *carpinifolia*	Females	2%	−46%	−34%	−20%	−70%	−48%
	Males	−36%	−5%	−29%	−47%	−31%	−84%
*Quercus* *robur*	Females	34%	−22%	−74.7%	−56%	−61%	−35%
	Males	−60%	−11%	−48%	−73%	−64%	−85%
*Quercus* *pubescens*	Females	35%	−27%	−48%	−56%	−62%	−52%
	Males	−50%	65%	−39%	−63%	−51%	−59%

Individuals with a null leaf consumption rate were removed from the dataset. A positive percentage indicated storage, whereas a negative value indicated a depletion of triglyceride content.

**Table 3:** Spearman correlations between the mean leaf litter consumption rates (mg.d^−1^.mg^−1^) for the five leaf litter species and the percentages of variation of the mean body triglyceride content compared with controls (in mg.g^−1^ dry weight) for southern and northern populations, with males and females pooled.

Leaf litter species and their origin	Geographical origin of gammarids populations	N	Spearman R	t(N-2)	*p*
***Alnus glutinosa*** ** (North/South)**	North	6	−0.94	−5.7	**0.0048**
	South	6	0.09	0.17	0.872
***Carpinus betulus*** ** (North)**	North	6	0.14	0.29	0.787
	South	6	0.54	1.29	0.266
***Ostrya carpinifolia*** ** (South)**	North	6	0.14	0.29	0.787
	South	6	−0.66	−1.74	0.156
***Quercus robur*** ** (North)**	North	6	0.54	1.29	0.266
	South	6	−0.20	−0.41	0.704
***Quercus pubescens*** ** (South)**	North	6	−0.37	−0.80	0.468
	South	6	−0.49	−1.11	0.329

Otherwise, the mean triglyceride content of females was significantly higher than that of males in the northern (+64%; F _1,243_  = 20.8, *p*<0.001) and southern (+33%; F _1.182_  = 29.6, *p*<0.001) populations.

## Discussion

In this study, we tested whether Mediterranean leaf litter species adapted to dry environmental conditions would show higher leaf toughness than northern leaf litter species and would be less consumed by gammarids. We tested whether southern populations of *G. pulex* would better consume tougher leaves and therefore consume more southern leaves than the northern populations of G. *pulex*. Finally, we tested the existence of a correlation between the amount of food consumed and the efficiency of energy storage in animals.

### Comparison between northern and southern leaf species

The initial (i.e., prior to leaf conditioning by aquatic fungi) thickness and toughness values showed differences between leaf species. Southern *Q. pubescens* leaves were thicker and tougher than northern *Q. robur* leaves. In the Rhône River Valley, the southern leaf litters were derived from trees adapted to the Mediterranean climate, whereas the northern leaf litters were derived from trees adapted to a continental climate. Hence, southern leaves are more likely to be exposed to desiccation [53], [54] and adapted to harsh environmental conditions. The adaptation to drier conditions would result in the development of a cuticle and mesophyll tissues rich in tannins, phenols or wax components [31], [55], [32], leading to a tougher structure than northern leaf litters. Nevertheless, the initial toughness of northern *C. betulus* leaves did not differ from that of southern *O. carpinifolia* leaves. *O. carpinifolia* leaves might have developed a different resistance strategy for desiccation, which does not entail tougher tissues, such as stomatal control, osmotic adjustment, or cell-wall storage [56]. Thus, our first hypothesis was partially validated, although leaf toughness might depend on the leaf species, despite literature showing that vegetation in a Mediterranean climate (lower mean annual precipitation and higher summer temperatures), such as the southern vegetation examined in this study, generally has more resistant tissues [29], [31].

We detected a strong effect of leaf type (i.e., soft, medium and tough leaves) on the fungal biomass at the end of the conditioning, as previously observed [27], but we did not observe any correlation with the origin of leaves (i.e., north or south) for the hornbeam or either oak species.

Concerning the leaf litter consumption, we did not obtain evidence that Mediterranean leaf species (*O. carpinifolia* and *Q. pubescens*) were less consumed than their continental counterparts (*C. betulus* and *Q. robur*), contrary to our expectations in the first hypothesis concerning the effect of the geographical origin of the leaf litter on their consumption by gammarids. We did not measure differences both for the consumption rates and the leaf toughness between the soft *C. betulus* and *O. carpinifolia* leaves. However, oak leaves were consumed similarly by all gammarids, whereas *Q. pubescens* leaves were tougher than *Q. robur* leaves. These results do not support the correlation between leaf consumption rate and leaf toughness established by Foucreau et al. [27], suggesting that this correlation, observed when leaf species with contrasting toughness (i.e., soft, medium and hard) are considered, was not valid between tough leaf species, such as *Q. pubescens* and *Q. robur* in the present study. The lack of a strong correlation between leaf toughness and leaf consumption rate of closely related species might be obscured by differences in chemical composition or nutritional quality.

### Comparison of leaf consumption between northern and southern gammarid populations

Our results clearly showed that gammarids from southern populations consumed more leaf litter than northern populations, consistent with our second hypothesis. Moreover, the difference in consumption rates was amplified by the percentage of gammarids showing undetectable leaf consumption rates (barely 9% for southern gammarid populations *vs.* 66% for northern populations). One explanation might be that southern gammarids are better adapted to consume tougher leaves and therefore should be more efficient, in terms of leaf degradation, than northern gammarids. Another explanation might be that some gammarid populations from the south of the Rhône River Valley possess higher locomotory activities [18], which increase their energetic needs [57], [58]. Thus, higher energetic needs might explain an increase in food consumption to supply the energy demand (primarily associated with locomotor activity in gammarids [59].

The overall consumption rate pattern of all leaf species was similar between northern and southern gammarid populations, except for alder leaves, which were consumed the most by northern gammarid populations but were less consumed than hornbeam leaves by southern populations.

For hornbeam leaves (Mediterranean *vs*. continental) with intermediate toughness, we did not observe any differences in the consumption rate, regardless of the gammarid population. For oak leaves with a high toughness, the Mediterranean leaves of *Q. pubescens* were more consumed by southern gammarids than the continental leaves of *Q. robur*, although *Q. pubescens* leaves are tougher. This observation suggests that southern gammarids might be locally adapted to consume the Mediterranean leaves. Similarly, northern gammarids are adapted to the continental leaves of *Q. robur*, which are more consumed than the Mediterranean oak leaves.

These results show that potential adaptation to local trophic resources might influence the between-species changes in the leaf litter consumption rate. However, the possibility of local adaptation is only supported by the pattern of consumption of tough oak leaves and not by that of intermediate hornbeam leaves, potentially reflecting the fact that hornbeam leaves are not tough enough to involve any adaptation of gammarids to their consumption.

### Comparison of the effects of leaf litter species, gammarid population and gender on the triglyceride content

The triglyceride content generally decreased, but did not follow any clear pattern, regardless of the leaf species and the gammarid population considered. This result might reflect the fact that the leaf litter supply did not provide enough energy to maintain the triglyceride content in gammarids. Indeed, freshwater gammarid amphipods are often classified as omnivores, feeding on litter detritus, algae, fungi, and animals such as chironomid larvae [60], [61], [62], [47]. Possibly, a diversity of food is necessary to maintain energy stores (primarily triglycerides in gammarids [35]. Indeed, in the amphipod *Dikerogammarus villosus*, the variation in lipid content and growth rate of animals might be associated with variations in the food regime [35].

This study showed that the triglyceride content was not correlated with leaf consumption rate. Our third hypothesis is consistent with these data; the amount of consumed food is not necessarily associated with the efficiency of energy storage in animals [34], [35]. Thus, the relationship between leaf litter consumption rate and the triglyceride content in gammarids is more complex than a simple linear relationship. Dehedin et al. [44] showed that some other minor molecules constituting the body stores (mainly glycogen) were correlated with the leaf litter consumption rate of gammarids. However, the glycogen body content might only supply short-term energy demands (i.e., a rapid catabolism) [49], [51] and is probably less efficient for studying energy storage in relation to feeding activity over a 10-d experimental period.

The leaf consumption rate and the body triglyceride content of females were significantly higher than those of males, regardless of their geographical origin. Although we did not observe a correlation between the leaf consumption rate and the triglyceride content of animals, we observed that females consumed more leaves than males and showed a higher triglyceride content both at the beginning and the end of the experiment. One explanation might be that females lost fewer triglycerides than the males during the experiment. However, the mean percentage variation in triglyceride content in females (Table 3; 28±33%) for all leaf species was similar to that of males (Table 3; 36±35%). Another explanation might be that the ratio between the storage of feeding resources and the energetic needs for females is higher than for males. This gender effect is probably associated with the reproductive cycle of females, which is more costly than that of males [63], [64], [50].

## Conclusions

This study showed that the presupposed differences in toughness between Mediterranean and continental leaves are only true for oak leaves (i.e., tougher leaves). The weak differences in toughness between pairs of leaf species are consistent with the absence of a difference in the leaf consumption rate of gammarids. However, the leaf consumption rate of gammarid populations strongly differed according to their geographical origin; southern gammarids consumed considerably more litter than northern populations. Thus, the future efficiency of the litter degradation process along the Rhône River Valley and subsequent organic matter recycling, depends both on the potential northwards migration of southern gammarids and on the ability of northern ones to degrade the Mediterranean vegetation that will shift northwards over future decades. We also demonstrated that gammarid populations might be locally adapted to consume the toughest leaves, but no evidence was observed for less tough leaves. The modification of riparian vegetation in a climate change context might locally change leaf litter processing.

These results also showed that there is no clear correlation between leaf consumption rate and triglyceride stores in gammarids. However, these two parameters strongly varied between females and males, suggesting the consideration of gender for further studies.
